# Impact of case-based learning on critical thinking dispositions in Chinese nursing education: a systematic review and meta-analysis

**DOI:** 10.3389/fmed.2025.1452051

**Published:** 2025-03-17

**Authors:** Yunlu Xiang, Dong Liu, Liang Liu, I-Chun Liu, Lanka Wu, Hao Fan

**Affiliations:** ^1^School of Teacher Development, Chongqing University of Education, Chongqing, China; ^2^School of Physical, Anshan Normal University, Anshan, China; ^3^College of Humanities and Social Sciences, Yuan Ze University, Taoyuan, Taiwan; ^4^School of Tourism and Hotel Management, University of Sanya, Sanya, China; ^5^Chengdu JinNiu GuoTou TianFu Industry Investment Co., Ltd, Chengdu, China; ^6^School of Tourism and Service Management, Chongqing University of Education, Chongqing, China

**Keywords:** case-based learning, critical thinking, nursing education, impact, meta-analysis

## Abstract

**Background:**

Case-based learning (CBL) is recognized for its potential to enhance critical thinking in nursing education. This meta-analysis aimed to assess the impact of CBL alone or in combination with other methods on improving critical thinking dispositions among nursing students in China.

**Methods:**

A systematic search was conducted in databases including PubMed, Embase, Cochrane Library, CINAHL and China National Knowledge Infrastructure from inception of the databases through June 1, 2024. Studies that utilized the Chinese Version of Critical Thinking Dispositions Inventory (CTDI-CV) and compared CBL with traditional teaching methods were included. Random-effects models were used to pool the mean differences (MD) in critical thinking scores, and subgroup analyses were performed based on participant types and intervention methods.

**Results:**

Thirteen studies involving 1,396 participants were included. The pooled results indicated a significant improvement in critical thinking dispositions (MD = 26.39, 95% CI: 18.71 to 34.06). Subgroup analysis revealed that nursing interns and combinations of CBL with problem-based learning (PBL) reported higher improvements. Secondary outcomes showed significant gains in both theoretical knowledge and operational skills, with heterogeneity observed across studies (*I*^2^ > 79%). The Egger’s test (*p* = 0.95) suggested no significant publication bias.

**Conclusion:**

CBL significantly enhances critical thinking among nursing students in China, particularly when integrated with PBL. Despite the observed heterogeneity, the findings support the incorporation of CBL into nursing curricula to foster critical analytical skills. Further research should explore the contextual factors that affect the variability in outcomes.

## Introduction

Case-based learning (CBL) is a form of education that is scenario-based, whether from natural or simulated backgrounds, to enable students to solve problems and make independent decisions. This approach relates theoretical learning to practical challenges to enhance the development of analytical skills and an individual’s critical thinking dispositions, rather than focusing solely on critical thinking ability. Critical thinking dispositions refer to an individual’s attitude and mindset toward critical thinking, characterized by a willingness to reflect on problems, question existing assumptions, and explore different perspectives. It emphasizes whether students are motivated to actively engage in thinking and seek multiple solutions, laying the foundation for them to exhibit critical thinking ability when facing complex situations. In contrast, critical thinking ability refers to the cognitive skills involved in analyzing, evaluating, and reasoning in practical situations. While critical thinking ability is crucial, without a strong critical thinking disposition, students may lack the motivation to engage deeply in the thought process (1, 2). CBL, through its situational learning approach, effectively stimulates students’ critical thinking tendencies, supports the development of their critical thinking abilities, and enhances their clinical judgment and decision-making skills in fields such as medicine and nursing (3).

The primary role CBL plays in the promotion of critical thinking is essential in healthcare education since many decisions have to be made with much speed coupled with accuracy. As research states, CBL enhances students’ capabilities to evaluate clinical evidence and illuminates the art of making crucial decisions speedily. For instance, CBL leads to an enhanced understanding of the biological processes and clinical reasoning for medical students (4). Additionally, CBL can help biochemistry students connect theoretical concepts to real-world applications, such as understanding diabetes or cancer through enzyme function and metabolic pathways, while fostering critical thinking dispositions by encouraging them to question assumptions, evaluate evidence, and consider multiple perspectives (5, 6). Another meta-analysis presented significant improvements in critical thinking ability in nursing students (7).

Educators frequently combine CBL with other methods in educational practices, such as problem-based learning (PBL), particularly in clinical practice teaching (8). PBL is another student-centered teaching approach where students actively acquire knowledge and skills by solving open-ended, complex real-world problems. It emphasizes both independent research and reflection on the issues, as well as collaboration among students to share insights and solve problems together. This dual approach allows students to develop individual critical thinking skills while benefiting from collective problem-solving, fostering both independence and interdependence (9, 10). In nursing education, the integration of CBL’s scenario-based case analysis with PBL’s proactive problem-solving approaches helps comprehensively enhance students’ clinical reasoning, decision-making abilities, and practical skills, especially in promoting critical thinking. This integration was driven by educational reforms in Chinese nursing education, which sought to cultivate nursing professionals with high-level critical thinking and problem-solving abilities to address the complex and dynamic healthcare environment. CBL typically focuses on specific, well-defined cases with close-ended solutions, resulting in more predictable outcomes, and interventions are relatively short, aimed at quickly resolving particular problems. In contrast, PBL deals with open-ended problems, offering a broader range of potential outcomes, encouraging students to explore multiple solutions. PBL interventions tend to be longer, allowing for deeper exploration and the development of comprehensive solutions. Moreover, CBL involves narrow, closed solutions focused on immediate decision-making, while PBL promotes open-ended, multifaceted solutions, emphasizing collaboration and iterative problem-solving (11, 12). Research indicated that the combination of CBL and PBL not only improved nursing students’ clinical reasoning and decision-making skills but also enhanced their practical skills and theoretical application across various nursing specialties. Additionally, it significantly enhanced their critical thinking, enabling them to make more precise and effective clinical judgments in complex healthcare settings (13, 14).

In the complex and dynamic environment of healthcare, nurses must evaluate a broad spectrum of information and make decisions that are both timely and evidence-based. Critical thinking in nursing is connected to improved accuracy in diagnosis, fewer errors, and increased patient safety (15). It was reported that critical thinking is crucial in developing clinical judgment skills, which directly influence the quality of patient care (16).

In China, the initiation and adaptation of CBL in nursing education dawned out of broad educational reforms aimed at raising the quality and relevance of practice within medicine. Realizing the demand for graduates who would not only be equipped with knowledge but who would exhibit excellence in critical thinking and problem solving, educational policymakers and institutions have slowly infused CBL into their nursing curriculum. The application of CBL has proved to improve Chinese nursing students’ clinical reasoning and decision-making competence after implementation (17). Further, this CBL approach effectively develops practical skills and theoretical applications among the diverse nursing specialties (18).

Although CBL has enjoyed different recognized benefits in helping the students develop their critical thinking in nursing education, heterogeneity in previous findings may be contributed by various factors, including study design and assessment tools used and the educational context in which CBL was implemented. This meta-analysis aimed to synthesize all available research that can show the conclusive impact of CBL on critical thinking dispositions among Chinese nursing students. The results offer more conclusive evidence in identifying factors influencing the effectiveness of CBL.

## Methods

### Study design

This systematic review was written and reported according to the Preferred Reporting Items for Systematic Reviews and Meta-Analyses (PRISMA) checklist. The protocol has been registered on the international prospective register of systematic reviews under registration number CRD42024559890.

### Information sources and search strategy

Literature search was carried out in electronic databases as follows: PubMed, the Cochrane Library, Embase, CINAHL, and China National Knowledge Infrastructure (CNKI) from inception of the databases through June 1, 2024. We searched Medical Subject Headings (MeSH) terms and keywords including “Case-Based Learning,” “critical thinking,” “nursing education,” and “China.” Where necessary, we used Boolean operators (“AND,” “OR”) to refine the search. This strategy was customized according to the indexing and search features of each database in order to optimize the yield of possibly relevant studies (Supplementary Table S1).

### Inclusion and exclusion criteria

Only studies that met all the following conditions were included: (1) clinical controlled trial conducted in the context of nursing education in China; (2) the study subjects included nursing students, including those who were attending on-campus nursing programs and nursing interns who were undergoing clinical training; (3) use CBL alone or in combination with other methods, such as PBL, as intervention method; (4) adopted the Chinese Version of the Critical Thinking Dispositions Inventory (CTDI-CV) as the instrument for measuring initial dispositions of critical thinking (19). Experimental studies as well as observational studies were considered, provided quantitative outcomes were reported.

Exclusion criteria were as follows: (1) studies that did not measure CTDI-CV; (2) studies that did not use CBL as an intervention or focused on populations other than nursing students; (3) studies written in languages other than English or Chinese; (4) reviews, commentaries, studies lacking primary data, or those without full-text access; (5) studies that did not explicitly state the type of control condition or were not evaluated using pre-and post-intervention measures with CTDI-CV.

### Data extraction

We developed a standardized data extraction form to systematically collect the study identifier (author, year), sample size, characteristics of the participant (age range and gender distribution), and detailed information of the participants such as student level and specific nursing program. Data regarding the intervention, i.e., type and setting of CBL, and duration of intervention, were recorded. Two reviewers independently extracted data to minimize errors and enhance reliability. Differences between reviewers were resolved by discussion or by consulting with a third reviewer as needed.

### Outcome measurement

Primary outcome of our meta-analysis was the CTDI-CV score. The California Critical Thinking Disposition Inventory (CCTDI) uses the Delphi Report’s consensus definition of critical thinking as a theoretical basis to measure critical thinking disposition (20). The CTDI-CV is a Chinese adaptation of the CCTDI, specifically developed to assess critical thinking disposition within the context of Chinese culture (21). It is a standardized 70 item multiple-choice test that includes seven dimensions of CT inclination, including “seeking truth,” “open mindedness,” “analytical,” “systematic,” “confident,” “curious,” and “cognitive maturity.”

Theoretical and operational scores were secondary outcomes of our study. Theoretical scores were obtained through paper-based exams (multiple-choice and open-ended questions). Operational scores assessed the nursing services provided by students to patients and were rated by supervising instructors based on the students’ performance in clinical practice. The highest scores for both theory and operation were 100 points.

### Quality assessment

The Cochrane risk of bias in the eligible trials was evaluated using the Cochrane Bias Risk Tool (RoB2). This tool evaluates domains such as randomization processes, interventions that deviate from expectations, missing outcome data, measurement of outcomes, and selection of reported outcomes. RoB2 provided an overall evaluation of each trial, classifying the trials as having low risk of bias, high risk of bias, or some concerns (22). Any disagreements between the assessors were addressed through discussion or, in case consensus was not achieved, a third, independent adjudicator was involved for the final decision.

### Statistical analysis

Using Stata 16.0 (StataCorp LLC) for statistical analysis, which provided advanced capabilities for managing, analyzing, and graphing complex data sets. A random-effects model employing the DerSimonian-Laird method was utilized to aggregate the effect sizes across studies, accounting for potential heterogeneity among study results. The choice of a random-effects model was guided by the anticipated variability in study settings and intervention types. Heterogeneity was evaluated using the I^2^ statistic, and tau^2^ to estimate the variance between studies. Subgroup analyses were performed to explore differences in effects based on study characteristics, such as participant type and intervention method. Further, publication bias was assessed using Egger’s test, ensuring the robustness and reliability of the findings. Confidence intervals were set at 95% for all effect size estimates, and *p*-value greater than or equal to 0.05 was considered statistically insignificant.

## Results

### Study selection

**Figure 1:**
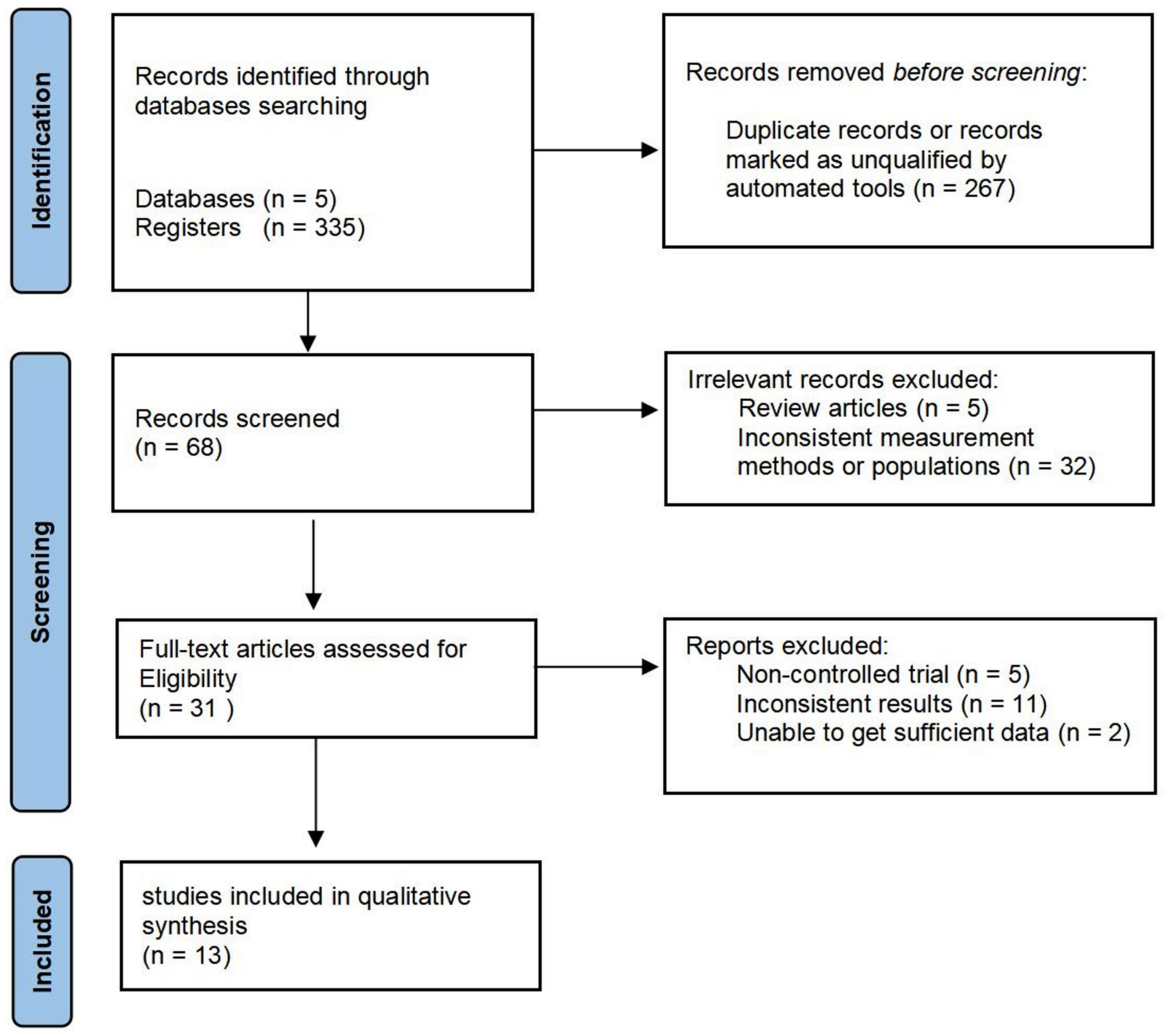
Flow diagram of study selection.

A total of 335 studies were identified in the initial search. 267 of these were excluded due to duplication or non-adherence to the inclusion criteria. These studies were then rigorously screened, resulting in the exclusion of 37 studies deemed unrelated to the research question, including 5 review articles and 32 studies with inconsistent measurement methods or populations. The remaining 31 full-text articles were thoroughly assessed for eligibility; criteria for exclusion included studies being non-controlled trials (*n* = 5), those reporting inconsistent results (*n* = 11), and articles from which sufficient data could not be obtained (*n* = 2). After this careful evaluation (Figure 1), 13 studies met all the inclusion criteria and were selected for qualitative synthesis (23–35).

### Characteristics of included studies

The 13 studies included in the meta-analysis were conducted between 2010 and 2022 across China, involving a total of 1,396 participants, primarily undergraduate students. These studies assessed the effectiveness of CBL, often combined with Problem-Based Learning (PBL), in enhancing critical thinking dispositions. Three studies did not report the age of participants, while the age range of participants in the remaining ten studies was between 19 and 23 years. Nine studies reported different gender distributions, while four studies did not provide gender information for participants. Most studies have compared CBL interventions with traditional teaching methods, where teachers primarily teach students in the form of lectures. The duration of these interventions varied from 2 months to 18 months, with multiple case scenarios being used during this time span (Table 1).

**Table 1:** Characteristics of included studies.

Study	Sample size	Age		Gender (M/F)	Participants	Intervene		Duration of intervention
		CBL group	Control group			CBL group	Control group	
Chen et al. (23)	46	mean (sd), 22.8 ± 0.7		10/36	Nursing interns	CBL + PBL	Traditional teaching	10 months
Qiao et al. (24)	49	mean (range), 21.4 (20–23)		0/49	Nursing interns	CBL + PBL	Traditional teaching	8 months
Wang et al. (25)	135	NR		NR	Nursing students on campus	CBL + PBL	Traditional teaching	6 months
Chen et al., (26)	154	mean (sd), 22.3 ± 1.2	mean (sd), 22.1 ± 1.1	5/149	Nursing interns	CBL + PBL	Traditional teaching	10 months
Hong and Yu (27)	122	Range, 20–22		NR	Nursing students on campus	CBL	Traditional teaching	8 months
Jia et al. (28)	83	NR		NR	Nursing students on campus	CBL + PBL	Traditional teaching	12 months
Zhang et al. (29)	120	Range, 21–23		9/111	Nursing interns	CBL + PBL	Traditional teaching	12 months
Li et al. (30)	80	NR		NR	Nursing students on campus	CBL	Traditional teaching	18 weeks
Zhu et al. (34)	87	mean (sd), 20.9 ± 2.2		15/72	Nursing students on campus	CBL + STEM	Traditional teaching	16 weeks
Song and Lu (31)	100	mean (sd), 22.9 ± 4.2	mean (sd), 23.0 ± 4.3	3/97	Nursing interns	CBL + PBL	Traditional teaching	12 months
Yu et al. (32)	295	mean, 19.9	mean, 20.1	35/260	Nursing students on campus	CBL	Blended learning	12 months
Zhai et al. (33)	90	mean (sd), 20.3 ± 1.1	mean (sd), 20.2 ± 1.1	0/90	Nursing interns	CBL + PBL	Traditional teaching	2 months
Ma et al. (35)	115	mean (sd), 20.2 ± 0.8	mean (sd), 20.6 ± 0.7	22/93	Nursing students on campus	CBL	Traditional teaching	12 months

CBL, case-based learning; PBL, Problem-based learning; STEM, science, technology, engineering, and math; CTDI-CV, Chinese Version of Critical Thinking Dispositions Inventory.

### Quality assessment

**Figure 2:**
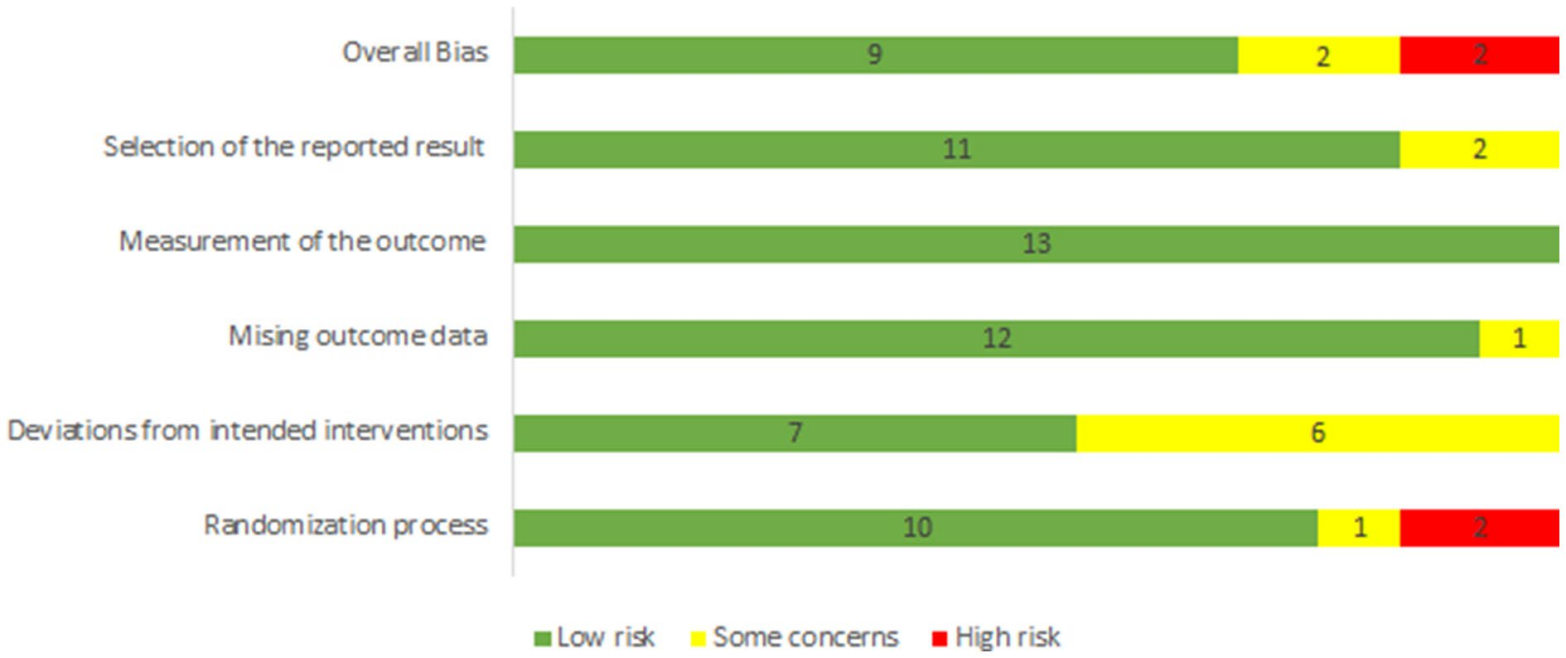
Risk of bias summary.

The assessment revealed a mixed level of bias across the studies. Notably, a few studies were judged to have a high risk of bias in several categories, particularly in the measurement of outcomes and selection of reported results. Conversely, several studies were rated with low risk in areas like randomization process and deviations from intended interventions (Figure 2; Supplementary Figure S1).

### Overall scores of CTDI-CV

**Figure 3:**
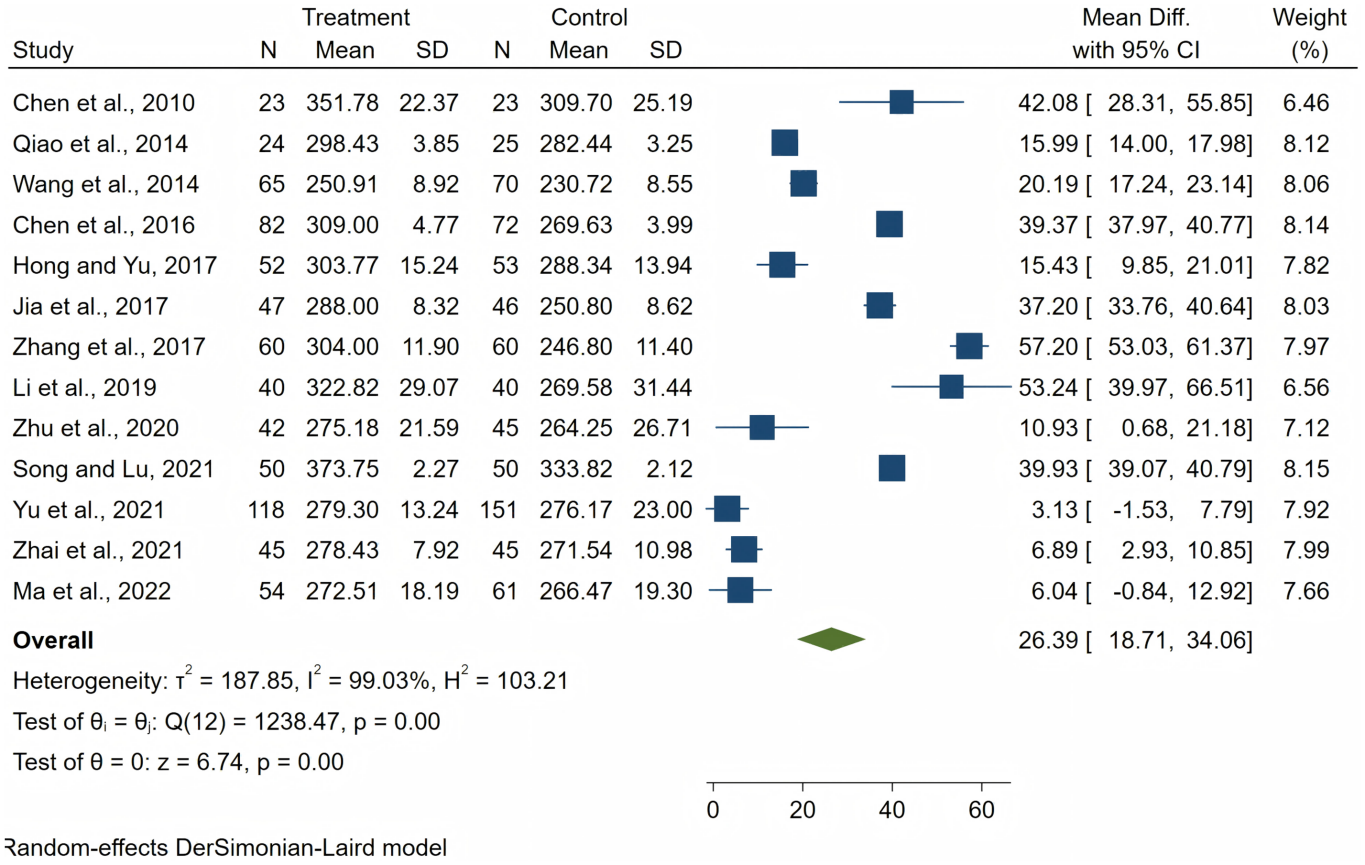
Forest plots of critical thinking dispositions.

**Figure 4:**
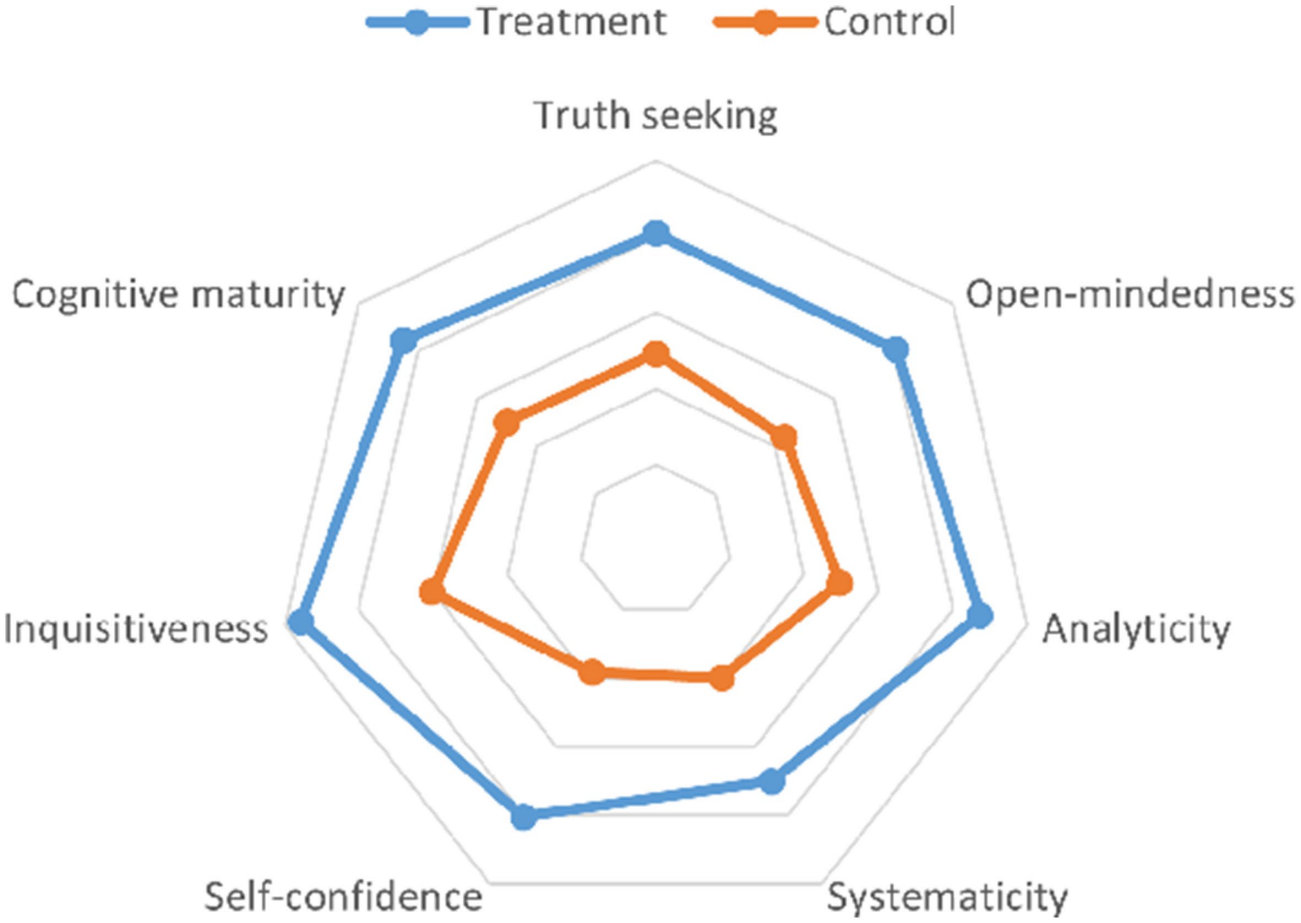
Radar chart of the seven configuration subscales of CTDI-CV.

Employing a random-effects model due to substantial heterogeneity (*I*^2^ = 99.03%, tau^2^ = 188.42), the analysis revealed a significant overall mean difference (MD) in critical thinking scores of 26.39 (95% CI 18.71 to 34.06). Individual study contributions varied, with MD ranging from a low of 3.13 to a high of 57.20, reflecting the diverse impact of CBL across different educational settings and study designs. The significant test of overall effect (*z* = 6.74, *p* < 0.01) confirmed the efficacy of CBL in enhancing critical thinking dispositions, despite the observed high heterogeneity among the studies included (*I*^2^ = 99.03%) (Figure 3). Figure 4 visually represented the comparative analysis, with each vertex corresponding to a different dimension of critical thinking: truth seeking, open-mindedness, analyticity, systematicity, self-confidence, inquisitiveness, and cognitive maturity.

### Subgroup analyses

**Figure 5:**
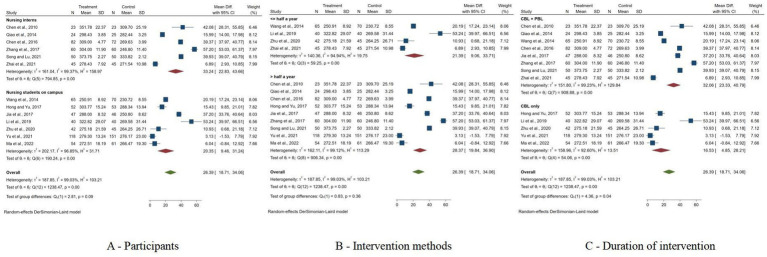
Forest plots of subgroup analysis results. **(A)** Forest plots of subgroup analysis results based on participants; **(B)** Forest plots of subgroup analysis results based on intervention methods; **(C)** Forest plots of subgroup analysis results based on duration of intervention.

The Subgroup analysis revealed that nursing interns experienced a higher improvement (MD = 33.24, 95% CI: 22.83 to 43.66) compared to on-campus nusring students (MD = 20.35, 95% CI: 9.46 to 32.24), although the heterogeneity within these subgroups remained high. When comparing intervention methods, studies incorporating both CBL + PBL showed a higher effect (MD = 32.06, 95% CI: 23.33 to 40.79) than those with CBL only (MD = 16.53, 95% CI: 4.85 to 28.21), with the difference between groups being statistically significant (*p* < 0.05). Furthermore, interventions lasting longer than half a year yielded a slightly higher effect (MD = 28.37, 95% CI: 19.84 to 36.90) compared to shorter durations (MD = 21.39, 95% CI: 9.06 to 33.71), although this was not statistically significant (*p* = 0.36) (Figure 5).

### Publication bias

The Egger’s test (*p* = 0.95) suggested no significant publication bias.

### Secondary outcomes

**Figure 6:**
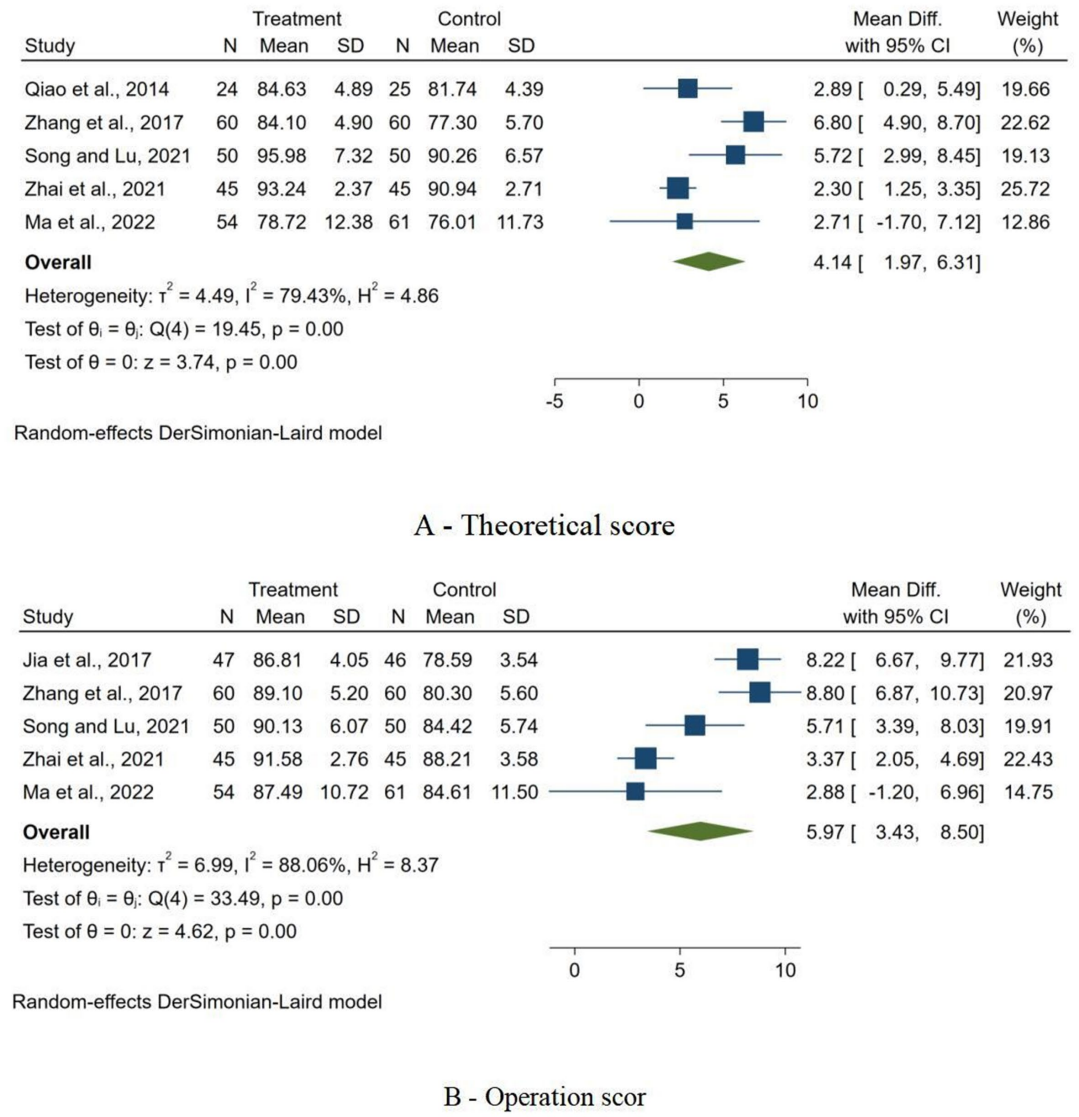
Forest plots of secondary outcome. **(A)** Forest plot of the impact of interventions on theoretical scores; **(B)** Forest plot of the impact of interventions on operation score.

The meta-analysis evaluated the effects of interventions on two distinct secondary outcomes (Figure 6). For theoretical score, the pooled MD was 4.14 (95% CI 1.97 to 6.31), despite high heterogeneity (*I*^2^ = 79.43%). For operation score, the meta-analysis yielded a MD of 5.97 (95% CI 3.43 to 8.50, *I*^2^ = 88.06%). Both outcomes confirmed the effectiveness of the interventions, with z-scores of 3.74 and 4.62 respectively, both highly significant (*p* < 0.01).

## Discussion

The meta-analysis revealed significant improvements in critical thinking dispositions among nursing students in China when engaged with CBL, as quantified by CTDI-CV, with an overall pooled MD indicating notable efficacy. Despite these positive outcomes, the analysis exhibited substantial heterogeneity, which might reflect variations in CBL implementation or contextual differences across studies. Subgroup analyses further highlighted that interventions were more effective among graduate students and when CBL was combined with PBL, suggesting that tailored approaches could enhance learning outcomes. Additionally, secondary outcomes of the analysis showed significant improvements in both theoretical knowledge and operational skills, reinforcing the multifaceted benefits of CBL. The Egger’s test suggested no significant publication bias, suggesting that the results were robust.

The findings are consistent with global educational trends that emphasize experiential and CBL approaches. It was reported that CBL effectively promotes self-directed learning and motivation in healthcare education, reflecting a broader impact on educational yields. The versatility of CBL was emphasized in fostering clinical competencies across diverse medical and healthcare disciplines, underscoring its effectiveness not only in knowledge acquisition but also in applying this knowledge to practical, patient-centered outcomes (36–38). However, the empirical evidence on its superiority over traditional learning methods in improving critical thinking or clinical skills remains inconclusive. The educational success of CBL may depend significantly on how it is implemented—emphasizing the role of interactive, student-centered learning environments that connect theoretical knowledge with practical application (39, 40).

The adaptability and effectiveness of CBL presented the efficacy across different healthcare education fields. Significant improvements were observed in knowledge acquisition, skill development, and comprehensive ability scores among dental students, psychology courses, as well as radiology education (41–43). Although these studies primarily focused on medical students, their findings were of significant relevance to nursing education because both share similar learning models and clinical skill applications (44). The research showed that CBL, by placing learning in real medical contexts, enhanced student engagement, thereby bridging the gap between classroom learning and practical clinical application. Moreover, CBL helped nursing students prepare for modern team-based care environments by emphasizing collaborative problem-solving and communication skills, which are essential for effective interdisciplinary teamwork. This approach allowed for tailored educational experiences that catered to the specific needs of nursing students, promoting self-directed learning and reflective practices crucial for lifelong learning and professional development. Importantly, CBL not only improved nursing students’ academic performance and case analysis skills but also increased their satisfaction and confidence in handling clinical tasks (45–47).

Nursing students are in a crucial phase, transitioning from classroom learning to professional practice. Campus-based nursing students focus mainly on theory, while nursing interns gain hands-on experience in clinical settings (30). The subgroup analysis showed that CBL was more effective for nursing interns. This may be due to their practical experience, which enhances clinical skills and helps them apply theory in real situations. Additionally, nursing interns receive immediate feedback from experienced staff, which accelerates their development and helps them manage complex situations better (31). However, the substantial heterogeneity observed in the study outcomes indicated that these benefits might vary significantly across different educational settings, personal attributes, and specific internship programs (48). Additionally, combining CBL with PBL leverages the strengths of both pedagogical approaches, creating a more dynamic and immersive learning environment (49). CBL focuses on specific cases to enhance clinical decision-making, while PBL encourages a broader investigation of problems, fostering a more extensive exploration of theoretical knowledge and its application (37). This combination encourages students to not only learn from specific cases but also develop a robust approach to problem-solving and critical thinking across different scenarios. Some research supports this, showing that hybrid approaches in educational strategies can significantly enhance learning outcomes by providing varied learning stimuli and broader contextual understanding (50, 51)

Although a large amount of information was generated from scratch in this meta-analysis, we ensured the rigor and transparency of the systematic review process by utilizing authoritative databases, such as PubMed, Embase, and the Cochrane Library, to ensure comprehensive coverage of relevant studies. To maintain methodological quality, we adhered to the PRISMA guidelines, following a structured framework for study selection, data extraction, and statistical analysis, ensuring that each step was clear and reproducible. Additionally, we employed RoB2 to assess the potential for bias in the included studies, evaluating key domains such as the randomization process, deviations from intended interventions, and handling of missing data. The combination of these tools not only enhanced the transparency of the review process but also ensured that the findings are based on high-quality, reliable evidence, providing a solid foundation for future research in this area. The future of CBL in nursing education looks promising, with a clear trajectory toward more integrated, interactive, and technologically enriched learning environments. As healthcare evolves, the demand for nurses who are not only clinically proficient but also capable of complex decision-making and problem-solving will escalate. CBL is expected to play a critical role in meeting these demands by further incorporating digital tools such as virtual reality and simulation technologies, which can provide realistic, immersive experiences that enhance learning outcomes (52). Additionally, there is a growing trend toward personalized learning, where CBL can be tailored to individual learning styles and needs, making education more effective and efficient (53). Future research should explore the scalability of CBL and its effectiveness in interdisciplinary education, promoting collaborative skills that mirror the real-world, team-based nature of healthcare (54). Such advancements will likely solidify the role of CBL in nurturing a more adaptive and resilient nursing workforce.

This meta-analysis has several limitations. First, the substantial heterogeneity across the included studies highlights the variability in CBL implementation and its contextual applications, which may limit the generalizability of the findings. Second, variations in research design, participant characteristics, and CBL implementation methods suggest that the effectiveness of CBL may differ significantly across educational settings, making it difficult to draw overarching conclusions. Third, the exclusive reliance on the CTDI-CV as the sole measure of critical thinking may not fully capture all dimensions of critical thinking that CBL aims to foster. Fourth, for some of the studies included in the literature, although their titles often mentioned “critical thinking ability,” the CTDI-CV used assessed individuals’ critical thinking disposition. This should be considered when interpreting the results. Finally, the relatively small sample size, focus on studies conducted in China, and reliance on a single measure of critical thinking, coupled with the exclusion of non-English and non-Chinese studies, limit the applicability of the results to other cultural and educational environments.

## Conclusion

This meta-analysis indicates that CBL is not only effective in improving critical thinking but also theoretical knowledge and operational skills, which is essential for clinical decision-making in nursing practice. Despite substantial heterogeneity among the included studies, which suggests that the effectiveness of CBL can vary significantly based on educational context and implementation methods, the overall positive outcomes support the integration of CBL into nursing curricula to cultivate critical analytical skills. Future research should focus on identifying the specific factors that contribute to the variability in effectiveness, potentially leading to more tailored and effective educational strategies.
