# Supplementary material for: Impact of case-based learning on critical thinking dispositions in Chinese nursing education: a systematic review and meta-analysis
Source: Front Med (Lausanne). 2025 Mar 17;12:1452051. doi: 10.3389/fmed.2025.1452051 (PMC11956162; doi:10.3389/fmed.2025.1452051)
Supplement: Supplementary file 1 [file Table_1.docx]

| **Database** | **Search Terms** |
| --- | --- |
| **PubMed** | ("Case-Based Learning" OR "CBL" AND ("critical thinking") AND ("nursing education" OR "nursing") |
| **Embase** | ('case based learning'/exp OR 'CBL') AND ('critical thinking'/exp OR 'critical thinking') AND ('nursing education'/exp OR 'nursing education') |
| **Cochrane Library** | (Title, Abstract, Keywords: "Case-Based Learning" OR "CBL") AND ("critical thinking") AND ("nursing education") |
| **CNKI** | ("基于案例教学" OR "CBL") AND ("批判性思维" OR "临床思维") AND ("护理教育") |
| **CINAHL** | (case-based learning or case-based reasoning or case based learning or case based reasoning) AND (critical thinking) AND (nursing) |

Table S1. search strategy
